# The projected increase of vertebral osteomyelitis in Germany implies a demanding challenge for future healthcare management of aging populations

**DOI:** 10.1007/s15010-024-02243-8

**Published:** 2024-04-09

**Authors:** Vincent Johann Heck, Tobias Prasse, Kristina Klug, Juan Manuel Vinas-Rios, Stavros Oikonomidis, Alexander Klug, Nikolaus Kernich, Maximilian Weber, Nicolas von der Höh, Maximilian Lenz, Sebastian Gottfried Walter, Bastian Himpe, Peer Eysel, Max Joseph Scheyerer

**Affiliations:** 1 Department of Orthopedics and Trauma Surgery, Faculty of Medicine and University Hospital Cologne, University of Cologne, Kerpener Str. 62, 50937 Cologne, Germany; 2 Center for Spinal Surgery, Sana Klinikum Offenbach, Starkenburgring 66, 63069 Offenbach, Germany; 3 Department of Neurological Surgery, University of Washington, Seattle, WA 98105-3901 USA; 4 Department of Psychology, Goethe-University Frankfurt, Theodor-W.-Adorno Platz 6, PEG, 60629 Frankfurt am Main, Germany; 5 Department of Trauma and Orthopedic Surgery, BG Unfallklinik Frankfurt am Main, Friedberger Landstr. 430, 60389 Frankfurt am Main, Germany; 6 Department of Orthopedics, Trauma Surgery and Plastic Surgery, University of Leipzig Medical Faculty, Leipzig, Germany; 7 Interdisciplinary Center for Spinal Surgery, St. Elisabethen-Krankenhaus Frankfurt, Ginnheimer Straße 3, 60487 Frankfurt am Main, Germany; 8 Department of Orthopedics and Trauma Surgery, Medical Faculty, University Duesseldorf, 40225 Duesseldorf, Germany

**Keywords:** Forecast model, Projection, Vertebral infection, Osteomyelitis, Spondylodiscitis

## Abstract

**Purpose:**

Since an increase in the occurrence of native vertebral osteomyelitis (VO) is expected and reliable projections are missing, it is urgent to provide a reliable forecast model and make it a part of future health care considerations.

**Methods:**

Comprehensive nationwide data provided by the Federal Statistical Office of Germany were used to forecast total numbers and incidence rates (IR) of VO as a function of age and gender until 2040. Projections were done using autoregressive integrated moving average model on historical data from 2005 to 2019 in relation to official population projections from 2020 to 2040.

**Results:**

The IR of VO is expected to increase from 12.4 in 2019 to 21.5 per 100,000 inhabitants [95% CI 20.9–22.1] in 2040. The highest increase is predicted in patients over 75 years of age for both men and women leading to a steep increase in absolute numbers, which is fourfold higher compared to patients younger than 75 years. While the IR per age group will not increase any further after 2035, the subsequent increase is due to a higher number of individuals aged 75 years or older.

**Conclusions:**

Our data suggest that increasing IR of VO will seriously challenge healthcare systems, particularly due to demographic change and increasing proportions of populations turning 75 years and older. With respect to globally fast aging populations, future health care policies need to address this burden by anticipating limitations in financial and human resources and developing high-level evidence-based guidelines for prevention and interdisciplinary treatment.

## Introduction

Vertebral Osteomyelitis (VO, syn.: spondylodiscitis) is a severe infection of the spine, and in recent decades, it has become more frequent [1–3]. However, despite wider availability of radiological imaging for the broader public and improvement of modern surgical techniques, VO remains life-threatening. Besides a mortality rate up to 17–23%, VO is associated with prolonged hospital stays, and has a high impact on a reduced quality of life in the long-term outcome [4, 5].

Due to demographical change, the population of developed countries is turning older on average, and the increasing total number of elderly individuals that are more prone to immunosuppression, cardiovascular diseases, and surgical-site infection is expected to cause even higher incidence rates of VO [6–9]. Moreover, increased use rates of reserve antibiotics due to emergence of multidrug-resistant bacteria, VO-associated infectious endocarditis, and other age-related complications will additionally challenge the treatment of those often multi-morbid patients [10, 11]. These developments will seriously challenge established health care systems within the next decades by requiring more human and financial resources.

In order to provide a reliable estimation of future native VO incidence, we forecasted the sex and age-related changes according to numbers and incidence of VO based on a nationwide projective analysis.

## Methods

### Study design and setting

The current population-based analysis is based on historical data from Germany's national hospital statistics, which is provided by the Federal Statistical Office, a German higher federal authority in the portfolio of the Federal Ministry of the Interior. It is the leading institution that guarantees independent quality-assured information and it is the only institution in Germany that documents, analyzes, and provides data on surgeries and diagnoses of full inpatients throughout Germany [12]. This database includes all annual inpatient treatment reports of all German hospitals and medical facilities since 2005, which legitimates this study as a nationwide survey (excluding military and psychiatric facilities). The data are based on the International Statistical Classification of Diseases and Related Health Problems, 10th edition (ICD-10). The ICD-10 was used to identify all cases of native VO. Such, all ICD codes ranging from "M46.20" to "M46.59" were extracted for data analysis. A detailed breakdown of these data by age and sex was made. For analysis, data were categorized by age into 12 groups: < 35, 35–39, 40–44, 45–49, 50–54, 55–59, 60–64, 65–69, 70–74, 75–79, 80–84, ≥ 85. The deadline of each year was December 31. All cases reported between 2005 and 2019 were included based on the corresponding ICD codes in their current version. No coding changes were made during the study period.

The population projections used were taken from the official statistics of the population projection until 2040 [13]. These population projections take into account future life expectancy, birth rates, and immigration rates. The model is based on a strong increase in life expectancy (life expectancy: men: 86.2 years, women: 89.6 years) and moderate birth rates (birth rate: 1.55 children per woman) and immigration rates (immigration rate: 221,000 per year).

### Projection methodology and statistical analysis

The statistical analysis was performed using „R “ (Version 2022.02.3) in accordance with the Guidelines for Accurate Health Estimates Reporting (GATHER statement) [14]. The historical data from 2005 to 2019 served as baseline years and population projections up to 2040 were then used to forecast the annual incidence of VO in Germany. We calculated the incidence by dividing the computed number of VO cases for the national total and for each age and sex subgroup by the corresponding official population projection. Based on previous prognostic calculations in the medical field, four alternative prognostic models were applied to evaluate and compare the predicted annual prevalence for VO in Germany: (Quasi-)Poisson regression (Poisson), logarithmic regression (Log), autoregressive integrated moving average modeling (ARIMA), and exponential smoothing (ETS) [15–18].

To estimate the annual number of VO via Poisson age, gender and calendar year were used as covariates. We adjusted the expected mean incidence for population size using the age-specific logged population numbers as offset to consider differences in prevalence between population subgroups as well as changes over time. Two-way interactions between age, sex, and calendar year were included in the regression model. Similar to Poisson, age, sex, and calendar year were included as covariates for Log. With ARIMA and ETS, we also used time series forecasting to project future numbers of VO [8, 19, 20]. ARIMA models tend to describe autocorrelation in the data, whereas ETS models are built on a description of trend and seasonality in the data and weight averages of past observations, with the weighting decreasing exponentially as the observations get older. In both cases, future values must be linear functions of past observations. In this study, grouped time series analysis was used to model time effects and to account for the respective sex and age groups.

All estimated rates and numbers of the models are provided with 95% confidence intervals (CI). We evaluated the predictive accuracy of each model using out-of-time cross-validation and thus dividing the dataset into training (years 2005–2014) and testing (years 2015–2019) subsets in a ratio of approximately 75:25, as recommended by Hyndman and Athanasopoulos, and comparing the mean absolute percentage error (MAPE), root mean square error (RMSE), mean absolute error (MAE), and the Akaike information criterion (AIC) of the models [21, 22] with the intention to choose the most robust model for our analysis.

## Results

### Historical data

In the time between 2005 and 2019, 115,283 VO cases were registered in Germany, of which 45,955 were diagnosed in patients aged 75 years or older. The total number of VO cases increased by 125.1% during this period with an average annual growth rate of 1.1. With respect to the population growth, the annual overall incidence changed substantially from 5.5 per 100,000 in 2005 to 12.4 per 100,000 inhabitants in 2019. In 2019, the most recent baseline year, a total of 10,306 VO cases were recorded in Germany, of which 230 VO were on multiple locations, 794 VO were on the cervical, 1906 VO on the thoracal, 542 VO on the thoraco-lumbar, and 6342 VO on the lumbar spine; 492 VO were not further specified. On average, VO was 1.4 times more often diagnosed in men than in women during the study period, whereas the average incidence rate of VO for individuals 75 years and older was 1.6 times higher in males than in females.

### Projections up to 2040

Based on the validation dataset, ARIMA model fit historical data best and appeared as most appropriate model. Thus, we selected ARIMA, which showed highly accurate forecasting, to project the future incidence of VO.

**Fig. 1:**
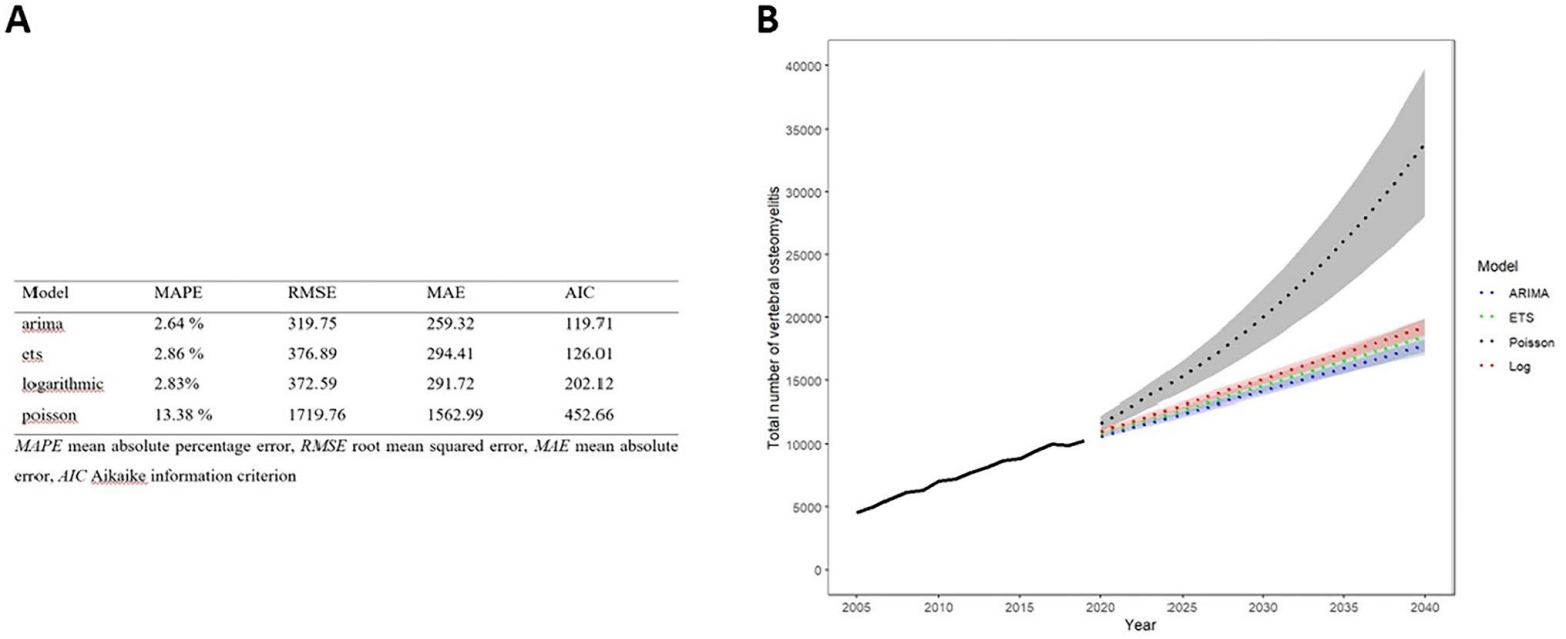
Accuracy criteria (**A**) and projected total number of VO (**B**; confidence intervals in shades) until 2040 depending on the model used. ARIMA model (autoregressive integrated moving average), ETS model (exponential smoothing), Poisson (Quasi-Poisson regression), and Log (logarithmic regression) are established forecast models. According to key performance indicators (MAPE, RMSE, MAE, AIC) that measure the accuracy of the different forecast models, ARIMA model is the most robust and provides high accuracy data

Despite a slight decrease in total population, ARIMA model predicts a substantial increase in the total number of VO cases of 72.6% to 17,789 [95% CI 17,304–18,274] in Germany in 2040, compared to 10,306 cases in 2019 (Fig. 1B).

**Fig. 2:**
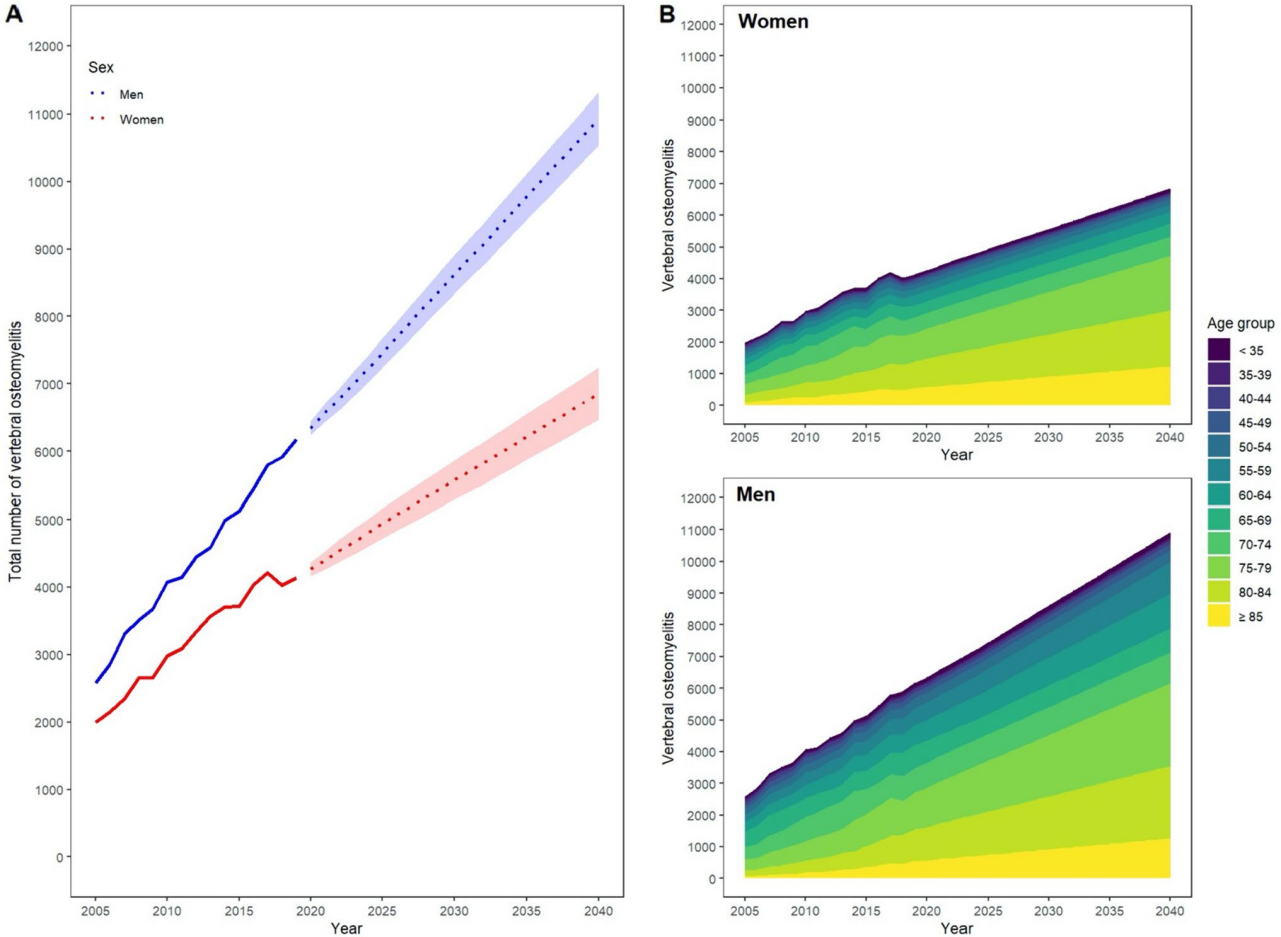
Projected total number of VO for women and men (**A**) in total: historical data (blue and red line) from 2005 to 2019 and projected numbers with confidence intervals in shades from 2022 to 2040 (**B**) and per age group from 2005 until 2040

The incidence of VO will increase from 12.39 in 2019 to 21.51 per 100,000 inhabitants [95% CI 20.92–22.09] in 2040 (Table 1). By 2040, the incidence rate of women with VO will increase by approximately 67.4% to an incidence rate of 16.42 per 100,000 [95% CI 15.50–17.35]. This corresponds to a total number of 6856 VO [95% CI 6470–7242] in 2040 up from 4134 in 2019. For men, the incidence rate is expected to increase by 77.5% to 26.69 per 100,000 [95% CI 25.74–27.64], corresponding to a total number of 10,933 [95% CI 10,545–11,320] VO cases in 2040 up from 6172 in 2019 (Fig. 2A).

**Table 1:** Predicted total numbers and incidence rates of VO for projection years in relation to 2019

Year	Absolute numbers	Relative increase [%]	Population	Incidence rate (per 100,000)	Relative increase [%]
2019	10,306		83,166,711	12,4	
2020	10,616 [10,489–10,742]	3.0 [1.8–4.2]	83,155,031	12.8 [12.6–12.9]	3.0 [2.0–4.2]
2021	10,973 [10,803–11,142]	6.5 [4.8–8.1]	83,514,000	13.1 [12.9–13.1]	6.0 [4.4–7.7]
2022	11,318 [11,114–11,522]	9.8 [7.8–11.8]	83,620,000	13.5 [13.3–13.8]	9.2 [7.2–11.2]
2023	11,677 [11,446–11,908]	13.3 [11.1–15.5]	83,701,000	13.9 [13.7–14.2]	12.6 [10.3–14.8]
2024	12,031 [11,776–12,286]	16.7 [14.3–19.2]	83,752,000	14.4 [14.1–14.7]	15.9 [13.5–18.4]
2025	12,395 [12,119–12,671]	20.3 [17.6–22.9]	83,759,000	14.8 [14.5–15.1]	19.4 [16.7–22.1]
2026	12,754 [12,459–13,049]	23.8 [20.9–26.6]	83,749,000	15.2 [14.9–15.6]	22.9 [20.0–25.7]
2027	12,117 [12,804–13,430]	27.3 [24.2–30.2]	83,711,000	15.7 [15.3–16.0]	26.4 [23.4–29.5]
2028	12,477 [13,147–13,807]	30.8 [27.6–34.0]	83,683,000	16.1 [15.7–16.5]	30.0 [26.8–33.1]
2029	13,834 [13,489–14,180]	34.2 [30.9–37.6]	83,638,000	16.5 [16.1–17.0]	33.5 [30.1–36.8]
2030	14,191 [13,830–14,551]	37.7 [34.2–41.2]	83,572,000	17.0 [16.5–17.4]	37.0 [33.5–40.5]
2031	14,548 [14,173–14,923]	41.2 [37.5–44.8]	83,515,000	17.4 [17.0–17.9]	40.6 [36.9–44.2]
2032	14,904 [14,515–15,293]	44.6 [40.8–48.4]	83,443,000	17.9 [17.4–18.3]	44.1 [40.4–47.9]
2033	15,262 [14,860–15,664]	48.1 [44.2–52.0]	83,367,000	18.3 [17.8–18.8]	47.7 [43.8–51.6]
2034	15,621 [15,206–16,036]	51.6 [47.5–55.6]	83,290,000	18.7 [18.3–19.3]	51.3 [47.3–55.4]
2035	15,981 [15,553–16,408]	55.1 [50.9–59.2]	83,213,000	19.2 [18.7–19.7]	55.0 [50.8–59.1]
2036	16,342 [15,902–16,781]	58.6 [54.3–62.8]	83,125,000	19.7 [19.1–20.2]	58.6 [54.4–62.9]
2037	16,703 [16,252–17,154]	62.1 [57.7–66.4]	83,028,000	20.1 [19.6–20.7]	62.3 [58.0–66.7]
2038	17,065 [16,602–17,528]	65.6 [61.1–70.1]	82,925,000	20.6 [20.0–21.1]	66.1 [61.6–70.6]
2039	17,427 [16,954–17,901]	69.1 [64.5–73.7]	82,823,000	21.0 [20.5–21.6]	69.8 [65.2–74.4]
2040	17,789 [17,304–18,274]	72.6 [67.9–77.3]	82,707,000	21.5 [20.9–22.1]	73.6 [68.8–78.3]

The corresponding 95% CIs are shown in parentheses. Incidence rate given per 100,000 individuals, relative increase in % compared to 2019 baseline data

**Fig. 3:**
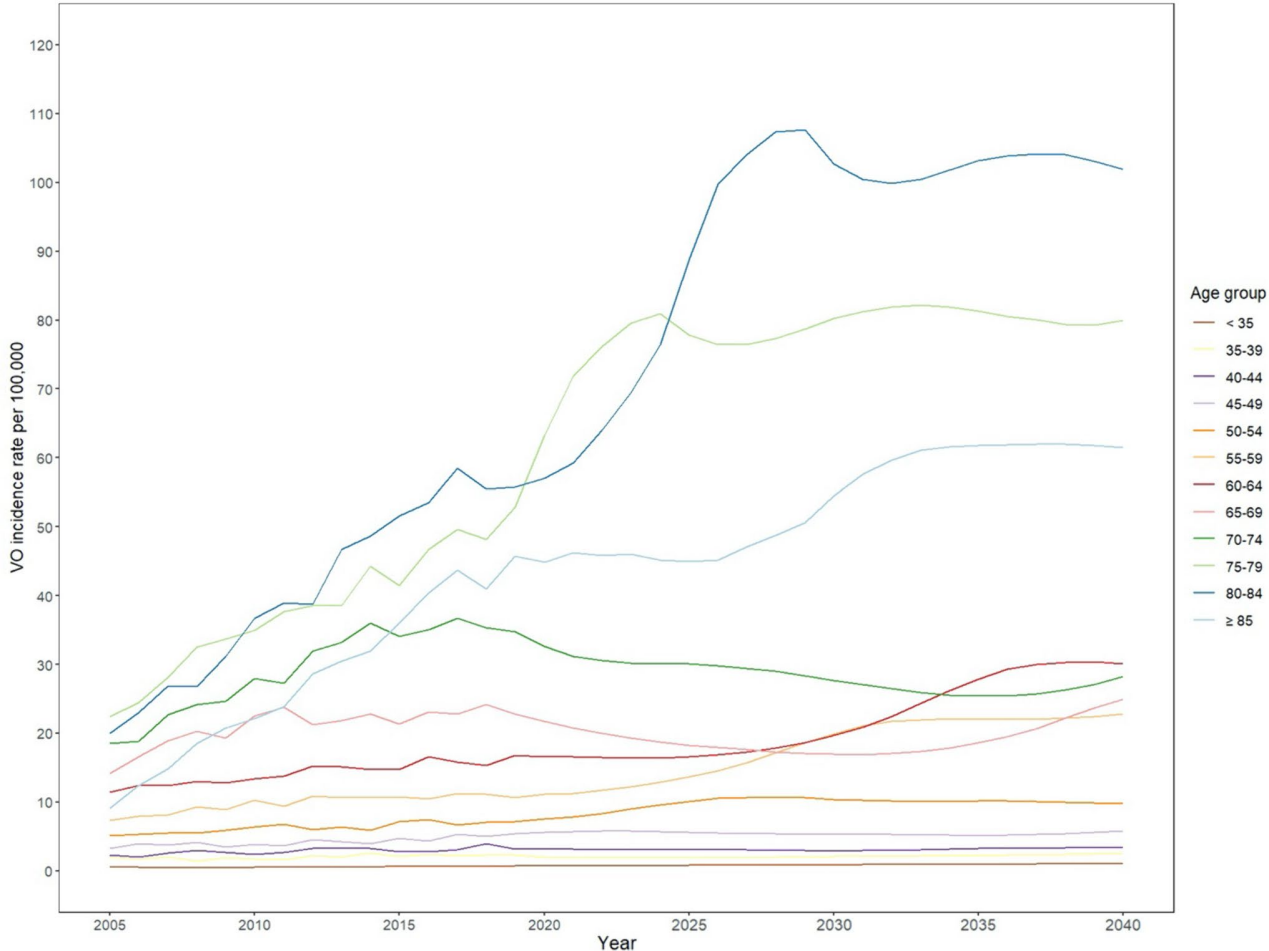
Projected incidence rates of VO per age group until 2040. The clinically relevant climax of incidence rate is projected between 2030 and 2035 for each age group. The largest incidence is shown for patients aged 75–79 years, 80–84 years, and ≥ 85 years

Analyzing data by age groups, there is a 118.5% increase in the number of absolute VO cases in patients aged 75 years and older compared to an increase of 29.7% in patients younger than 75 years. The age group with the most significant change in absolute numbers of VO will be patients 85 and older showing an increase of 128.6% until 2040. This trend applies for both women and men, with a 2.3-fold increase for women and men aged 85 years and older (Fig. 2B). By 2040, the incidence for VO will be the highest for patients aged 80 to 84 years (Fig. 3).

**Fig. 4:**
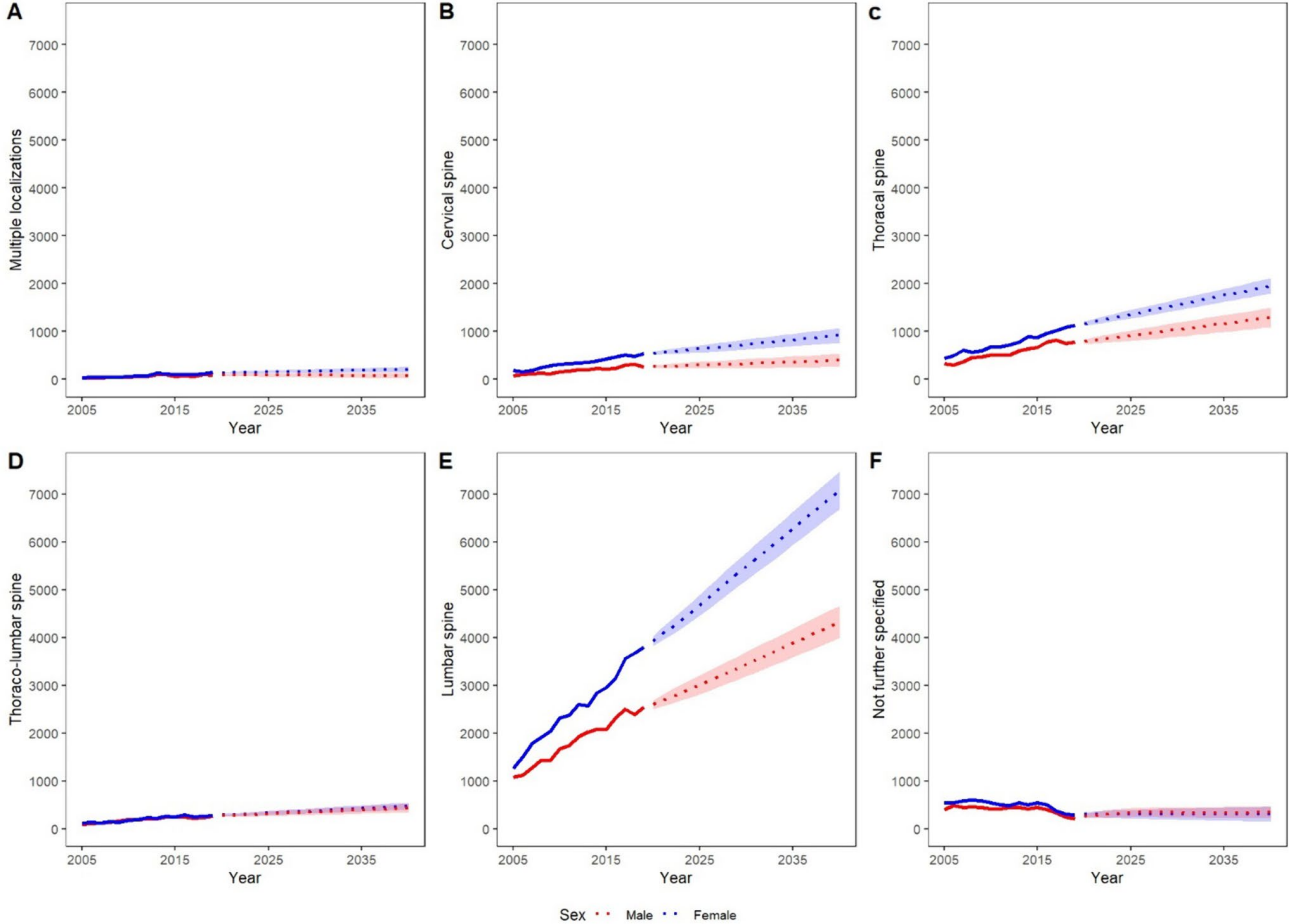
Past and projected total numbers of VO for men and women per localization on the spine until 2040. Abscissa and ordinate are scaled identically to provide better comparability between different levels. The highest incidence rate of VO is projected in the area of the lumbar spine for both women and men

The VO category with the greatest change in numbers will be the group of lumbar VO, which is expected to increase by 79.9% to a total number of 11,410 [95% CI 10,955–11,866; IR = 13.8] in 2040. Large increases can also be found in thoracal, thoraco-lumbar, and cervical VO with a rise of 70.1% from a total number of 1906 thoracal VO in 2019 to 3242 [95% CI 3013–3472; IR = 3.9] in 2040, of 66.8% from a total number of 542 thoraco-lumbar VO in 2019 to 904 [95% CI 801–1007; IR = 1.1] in 2040, and of 64.8% from a total number of 794 cervical VO in 2019 to 1309 [95% CI 1154–1463; IR = 1.6] in 2040. The smallest increases can be recorded in not further specified VO with a rise in total numbers of 20.9% and for VO at multiple localizations on the spine with a rise of 31.1%, corresponding to a total number of 645 [95% CI 476–814; IR = 0.8] not further specified VO in 2040 up from 492 in 2019 and of 278 [95% CI 205–351; IR = 0.3] VO at multiple localizations on the spine in 2040 up from 230 in 2019. Those changes in different VO categories apply to both, men and women (Fig. 4).

## Discussion

In this modeling study, we conducted a forecast analysis to approximate the future incidence of native VO as relevant burden of health care systems. We used cumulative data of 15 years in a cohort of more than 80 million women and men to project future age- and gender-related trends of this life-threatening infection.

Hereby, this modeling study provides some notable strengths. Most important, our projections are based on the most robust out of four established forecast models. Furthermore, the underlying database provides outright information on historical trends of native VO in a large, industrialized population. Thus, despite long-term forecasting, which can be extremely useful for research and resource planning, our projections show high accuracy in pure mathematical terms.

We found a continuous increase in incidence of VO with a steep increase in patients aged 75 years and older. By 2035, the age-adjusted incidence is projected to meet its climax. However, our modeling study predicts an ongoing increase of total incidence up to the year 2040, mainly driven by a demographic shift toward an aging population in Germany leading to a higher proportion of women and men aged 75 years and older.

This finding has important implications on healthcare systems and policymakers: higher age itself and age-associated diseases including diabetes, cardiovascular diseases, malignancy, immunocompromised status, and use of hemodialysis are strongly associated with higher need for surgical treatment of VO. These factors, however, are also associated with higher rates of adverse events, reduced quality of life, longer hospital stays and, finally, fewer return-to-home rates as well as increased mortality. In summary, this suggests a large increase of direct and indirect costs, leading to a serious socioeconomic challenge [23–25].

The importance of this finding is highlighted by the high co-prevalence of infective endocarditis (IE) and emergence of antibiotic-resistant bacteria [10, 26]. As the epidemiology of IE—characterized by high morbidity, mortality and costs—has changed in a similar proportion comparted to VO in recent decades, consequently, the predicted mounting numbers of VO in patients aged 75 and older also suggest rising incidence rates of IE that will additionally impact health care systems [27–32]. Thus, managing human and financial resources effectively, focusing on prevention-research, developing interdisciplinary treatment strategies and networks will be of utmost importance to tackle this upcoming burden in future.

It is commonly accepted that VO most often results from hematogenic seeding. Our findings assume that this upcoming burden is not only due to native VO because iatrogenic or post-surgical VO need to be considered as an additional challenge for future healthcare systems [33]. In 2004, Deyo et al. demanded a shift in research effort from “how to perform fusion to examining who should undergo fusion” [34]. Ever since, the use of spinal surgery has been rising worldwide and a further increase of spinal surgical procedures is projected [35]. The incidence of surgical-site infections (SSI) is 4.4% in patients undergoing instrumented surgery, and 1.4% in non-instrumented surgery [36]. This ultimately could result in approximately 30% more VO when considering iatrogenic causes [37]. However, it must not be forgotten that not all SSI cases can be considered as postsurgical VO. Superficial wound healing problem are also included in that cohort and do not necessarily lead to an infection of the vertebral body or the disk. However, given the rapidly increasing incidence of spinal surgery associated VO worldwide in the last decades, this emphasizes Deyos’ 20-year-old demand of need for reasonable decision-making when indicating spinal surgeries [38]. Thus, considering an aging population innovative strategy of prolonged conservative care and modern minimal invasive surgical procedures patients may be considered as key factors for minimizing the burden of iatrogenic VO, with decreased costs as a result [39–41].

Our model suggested that men will be more prone to VO than women, and the highest number of VO is projected to occur in men aged 75 and older. Because postmenopausal biological sex does not provide sufficient causality for differential incidence rates, gender and associated life-style should be evaluated as possible risk factors in future studies [42].

Although our forecast model is highly accurate in pure mathematical terms, and projected numbers come along with small CI and high predictive accuracy, we used historical incidence rates to build the projections. As it is not predictable whether historical trends of native VO may change in future due to advances in biologics, genetics, antibiotic treatment, or preventive strategies, long-term projections are more prone to overestimation. However, a clinically relevant scientific breakthrough—which may result in relevant decrease in incidence of VO—is not very likely to occur in the imminent future, and, moreover, establishing new algorithms is laborious. Thus, we believe our projections to be highly accurate for the upcoming decade, and possibly thereafter. For this study, historical data of the past 15 years have been used only, as data documentation was insufficient before. Therefore, the projected incidence rates made in this study should be verified in future, evaluating whether a significant discrepancy to projected VO incidence is observed.

Although the data are provided by the Federal Statistical Office of Germany, an official national institution that demands high accuracy of data input and coding to provide complete, nationwide information, we could not separate VO in between different or multi-resistant pathogens. Furthermore, due to insufficient data accuracy, we could not additionally forecast iatrogenic VO. However, our projections provide a base for future studies to settle this issue and, thus, to quantify the upcoming burden.

It remains questionable, whether the projected tendency can be transferred into other health care systems in analogy. Although historical trends of VO in Germany face a similar direction as in other large European and Asian countries, population development differs into two principal directions within several large nations. The population in the United States and in some European countries, such as the United Kingdom, France, and Scandinavia, continues to grow driven by high fertility and immigrations rates, while others, such as Germany, Italy, Russia, and most Eastern European countries, face a decline in population resulting from low birth and immigration rates leading to a demographic shift—seek additional pressure human and financial recourses because of a shrinking working population [43]. However, the proportion of people aged 75 and older will increase in many countries. This is both a European and global phenomenon, as within the next decades many populations worldwide are likely to follow this trend (43, 44). Thus, the projected burden of VO linked to a higher amount women and men aged 75 and older is likely to apply to many other healthcare systems of developed countries.

In general perception, native VO is widely underestimated. Given current trends, we project the climax of age-dependent incidence of VO to occur in about ten years from now, however, the total numbers will further continue to increase substantially due to a shift toward patients aged 75 and older, with resultant serious socioeconomic challenges for health care systems in fast aging countries. This emphasizes the need for widespread establishment of interdisciplinary treatment strategies based on high-level evidence-based guidelines, as well as appropriate financial and human recourse management to challenge this serious burden in light of a worldwide aging population in the next decades.

## Data Availability

All data is publicly available upon reasonable request at the Federal Statistical Office of Germany (Destatis).
